# A prospective cohort study of novel functional types of parathyroid glands in thyroidectomy: In situ preservation or auto-transplantation?

**DOI:** 10.1097/MD.0000000000005810

**Published:** 2016-12-30

**Authors:** Qiuxia Cui, Zhihua Li, Deguang Kong, Kun Wang, Gaosong Wu

**Affiliations:** Department of Thyroid and Breast Surgery, Tongji Hospital, Huazhong University of Science and Technology, Wuhan, P.R. China.

**Keywords:** classification, hypoparathyroidism, organ preservation solutions, parathyroid gland, thyroidectomy

## Abstract

The best method of preventing hypoparathyroidism after thyroidectomy is to keep parathyroid glands in situ. However, hypoparathyroidism still regularly occurs with the existing parathyroid classification system, and the incidence of permanent hypoparathyroidism has not been reduced. We created a novel system for classifying parathyroid glands that can guide parathyroid preservation in thyroidectomy.

We prospectively observed parathyroid glands using the new system in 218 neck surgeries, compared with 132 under the traditional system from January 2014 to September 2015 at a single clinic center. Briefly, we classified parathyroid glands as follows: Type A, no dependency on the thyroid; B1, partial blood supply from the thyroid but retains adequate blood supply after removal of the thyroid; B2, partial blood supply from the thyroid and becomes devascularized after the removal of the thyroid; B3, blood supply mostly from the thyroid; and C, blood supply completely dependent on the thyroid. The classifications were used to decide between in situ preservation or auto-transplantation.

The most common type of parathyroid gland was type B1 (53.77%), followed by type A (20.89%), which are the perfect categories for in situ preservation. Type B2 (17.52%) and type B3 (1.21%) have a chance to be kept in situ. For type C (6.61%), in situ preservation is impossible. When in-situ preservation is ruled out, parathyroid auto-transplantation is an alternative, with partial or total gland tissue, depending on the classification and the surgeon's discretion. Among the patients who were classified under the new system, 43.6% presented with transient hypoparathyroidism (symptoms lasting ≤6 months) after surgery, versus 42.4% in the old system, which was not a significant difference. However, permanent hypothyroidism (symptoms lasting >6 months) was not detected in the applied group, but in 3.0% of patients in the nonapplied group (*P* = 0.01).

Our novel functional nomenclature system for parathyroid glands can provide a guide for preserving parathyroid function. For certain types, such as type B2 and C, instead of being kept in situ, auto-transplantation of partial or total parathyroid tissue is a prudent choice to ensure continued function.

## 1 Introduction

Hypoparathyroidism is one of the most common complications of thyroidectomy. Thyroid diseases are common worldwide, and thyroidectomy is an important treatment. As such, the complications associated with thyroidectomy need careful attention. Hypoparathyroidism is of particular concern to physicians in China. It is defined as a low secretion of the parathyroid hormone (PTH) that results in a deficiency of blood calcium and causes discomfort. Permanent hypoparathyroidism—hypoparathyroidism persisting for more than 6 months—is a debilitating morbidity associated with thyroidectomy. Studies from the British Association of Endocrine and Thyroid Surgeons audit showed the incidence of transient and permanent hypoparathyroidism to be 27.4% and 12.1%, respectively, for patients undergoing thyroidectomy.^[1]^ Attempts are being made to find a reliable way of preserving parathyroid function after thyroidectomy. Currently, the accepted method is to preserve in situ the parathyroid glands that are affected during the surgical procedure. This way, auto-transplantation is all that is needed as a remedial measure when a parathyroid gland is cut off inadvertently. However, this approach is not without controversy; even if almost all the parathyroid glands are preserved in situ, hypoparathyroidism and even permanent hypoparathyroidism can still develop.^[2]^

Parathyroid auto-transplantation might be the best way to avoid permanent hypoparathyroidism in total thyroidectomy, despite an increase in short-term hypocalcemia.^[3]^ However, there is no consensus regarding this. The main issue is identifying and deciding the appropriate occasion when an autograft is needed. After several years of observation and modification, we have created a novel system for classifying parathyroid glands at the functional level, which can enable the preservation of parathyroid gland function.

In this study, we sought to implement this system in a large contemporary cohort of patients, describe the relative frequency of each type of parathyroid gland, and evaluate its potential impact on the surgical procedure and overall outcomes.

## 2 Materials and methods

We prospectively reviewed a collected database of all patients who underwent first-time total or near-total thyroidectomy in the Section of Surgery within the Department of Thyroid and Breast Surgery at Tongji Hospital, affiliated with the Huazhong University of Science and Technology, from January 2014 to September 2015. All the surgeries were performed by an experienced thyroid surgeon (GW), as described previously.^[4]^ This cohort represents all patients treated since the development and application of our nomenclature system. The study protocol was approved by the relevant ethics review boards of the Tongji Medical University and the patients enrolled in this study provided their informed consent. Patients who had a disease requiring total or near-total thyroidectomy for the first time with or without lymph node dissection in the neck were enrolled in this study. Patients were excluded from the study if they: (a) underwent reoperation for thyroidectomy; (b) had Graves’ disease (a known risk factor for postoperative hypocalcemia)^[5]^; (c) had hypoparathyroidism for other reasons; (d) had incomplete data or had been followed for less than 6 months; or (e) the parathyroid parenchyma could not be confirmed, even by frozen section during the operation.

The current technique to preserve parathyroid function and reduce the incidence of hypoparathyroidism involves preserving the parathyroid in situ; auto-transplantation is a remedial measure for inadvertently cut or devascularized parathyroid glands. However, the indications for auto-transplantation are controversial. In this study, based on our experience with thousands of thyroidectomies, we classified parathyroid glands into 3 main types to provide a guideline for thyroidectomy and other neck surgeries.

We observed parathyroid glands using the new system in 218 neck surgeries, compared with 132 under the traditional system. The traditional technique was to preserve parathyroid function and reduce the incidence of hypoparathyroidism involves preserving the parathyroid in situ; auto-transplantation is a remedial measure for inadvertently cut or devascularized parathyroid glands. New protocol: preserving them in situ where possible, but once they changed color or turned out to be type C, an autograft would be performed in the sternocleidomastoid muscle or brachioradialis muscle in the forearm. Types B2, B3, and C should be auto-transplanted partially or totally.

**Figure 1:**
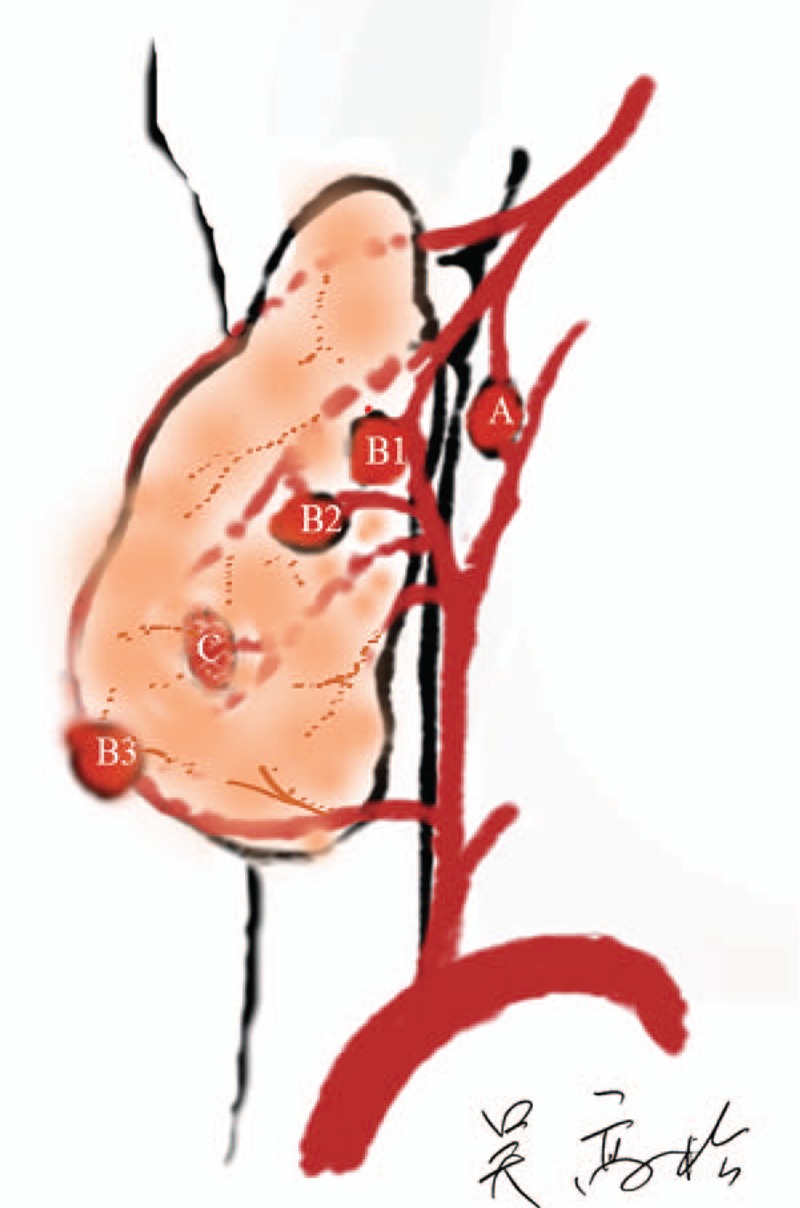
The 3 main types of parathyroid gland. From A to C: type A, nonattachment to the thyroid and has adequate blood supply; type B1, attached lightly to the thyroid and retains adequate blood supply after thyroid removal; B2, attached tightly to the thyroid and changes color easily, in which case, the distal tissue is cut in half for autograft; type B3, blood supply is derived mostly from the thyroid gland and may be treated as either type B2 or type C according to the surgeon's skill; type C, under cover of the thyroid capsule and can only be preserved by total auto-transplantation.

**Figure 2:**
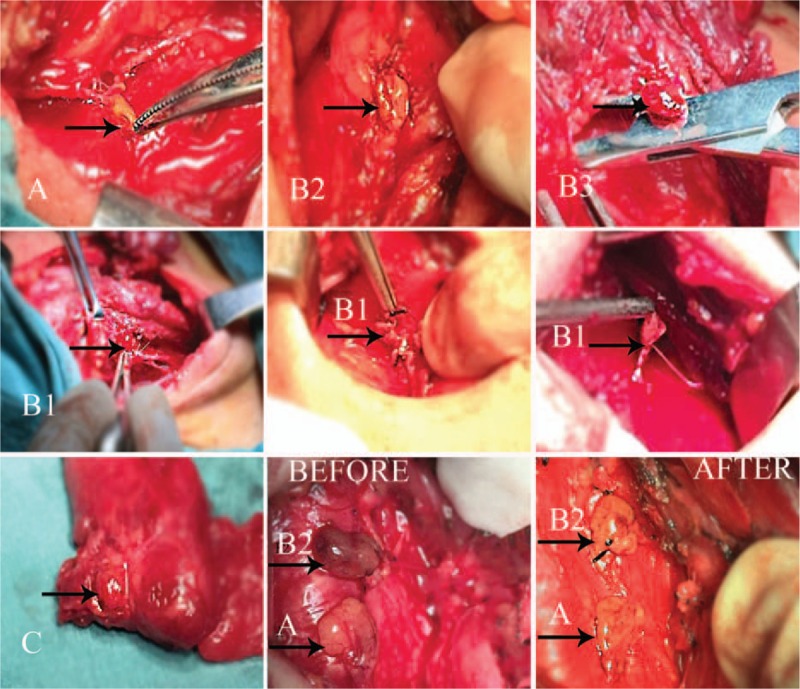
The virtual types of parathyroid glands in vivo: each type is shown respectively in the figure; the last 2 figures show the operation on a type B2 gland, before the performance of autograft for half of the distal part of the parathyroid tissue, its color is dark red once separated from the thyroid; after the partial autograft, the preserved type B2 gland changes color to pink.

This classification was performed in all patients in this study via intraoperative assessment and immediate classification by an experienced attending surgeon familiar with the nomenclature system. Our system is based on the relationship between the parathyroid gland and the thyroid gland as well as the color change in the parathyroid glands after separation from the thyroid, as illustrated in Figs. 1 and 2 (no significant symmetry difference in parathyroid anatomy between the left and the right side). The different categories of parathyroid glands are as follows:

Type A. Total nonattachment type (secure and thymus types): not attached to the thyroid gland, with adequate blood supply and no color change after thyroidectomy; requires meticulous attention in thyroidectomy with central lymph node dissection.

Type B. Partial dependency: needs to be screened

B1—attached to the thyroid gland but having adequate blood supply after separation from the thyroid; no color change after thyroidectomy.
B2—attached to the thyroid, with part of the blood supply derived from it; easily changes color during preservation. Once the color change is visible, the blood pressure of the parathyroid capsule should be reduced using scissors. If the color does not return to pink from dark red, an initiative auto-transplantation of the distal parathyroid gland should be performed to relieve the pressure burden of blood supply on the remaining tissue and to save the devascularized part.
B3—Blood supply mostly derived from the thyroid and located outside the thyroid capsule; difficult to preserve in situ but may have a chance to be preserved and treated as type B2. If it is not preserved, the gland will become type C and will require complete auto-implantation.

Type C: Complete attachment (type of risk, implant type): totally supplied by vasculature from the thyroid, often covered by the thyroid capsule and liable to be cut inadvertently. After inspection of the thyroid gland in vitro, auto-transplantation is required.

We routinely observed and evaluated the patients who underwent thyroidectomy (TT) with or without central lymph node dissection (CLND) and lateral compartment neck dissection (LCND), and recorded their parathyroid types according to the new classification.

While performing the classification, we paid attention to the following aspects: (a) what kind of parathyroid gland is found during the surgical procedure and how often each appears in the patients; (b) whether the patients suffer postoperative complications; (c) the symptoms of hypoparathyroidism that occurred and the relative treatments the patients received. During the operation, the parathyroid was imaged and classified, and the procedure of preserving the parathyroid glands followed our new protocol: preserving them in situ where possible, but once they changed color or turned out to be type C, an autograft would be performed in the sternocleidomastoid muscle or brachioradialis muscle in the forearm.

In China, instead of being discharged immediately after the operation, patients are typically discharged after several days, when they are fully recovered. Hence, we recorded the patients’ medical condition and the treatment administered to them for 1 week after surgery, while they remained in the hospital. Afterward, they were followed up via phone or during outpatient clinic visits for at least 6 months.

We recorded the number of cases where the parathyroid glands were preserved in situ, and those that underwent auto-transplantation. Previous studies have considered the surgical dissection time and the timing of discharge to be surrogates of the technical complexity of the surgery.^[6]^ However, the size of the thyroid and the presence of Hashimoto's disease or hyperthyroidism also affect the volume of bleeding and surgical dissection time. In addition, the patients were also observed for postoperative hypocalcemia. However, patients presenting with hyperthyroidism need calcium supplements for a long period; other diseases that affect the parathyroid function also affect blood calcium levels. For these reasons, those patients who presented with hyperthyroidism and parathyroid-related synchronous diseases derives from brain or other organs were excluded. Biochemical data at baseline, and at 3 days, 1 month, and 6 months’ post-operation were recorded for all patients by outpatient clinic and phone conversation. Most patients came to our hospital for reexamination and a blood test within the first postoperative year. Those who came with other hospitals’ test results or who rejected a blood test were excluded from the study.

Because the definition of transient and permanent (persistent) post-thyroidectomy hypocalcemia and hypoparathyroidism is controversial, we chose 6 months postoperatively as a cut-off time in the current study. This is in accordance with most reports.^[7]^ We also chose 6 months as an appropriate follow-up duration. Statistical analyses were performed using SPSS, version 13.0 (IBM, New York). Differences between categorical variables were assessed by the 2-sided χ^2^ test (Pearson's chi-square test), continuous variables were assessed by a 2-tailed Student's *t* test, and correlations were tested using the Spearman rank correlation test. Differences were considered statistically significant for *P* values <0.05.

## 3 Results

From January 2014 to September 2015, we performed thyroidectomies in 808 patients in our institution. Of these, 391 patients were included based on the inclusion and exclusion criteria. However, 237 patients of them were classified under the nomenclature system. Another 154 patients accepted the preservation of parathyroid glands using traditional methods and were not classified under our novel nomenclature. During the follow-up period, 20 patients opted out of the study, 8 lacked complete records, 8 patients with secondary hyperparathyroidism required continuous renal dialysis, and 5 patients received radioactive iodine therapy within 6 months, which has been reported to affect the postoperative recovery of parathyroid function. All of these 41 patients were excluded. Finally, we applied the novel system to 218 patients and included a further 132 patients as controls in the study. The clinical characteristics of the study sample are presented in Table 1.

**Table 1:**
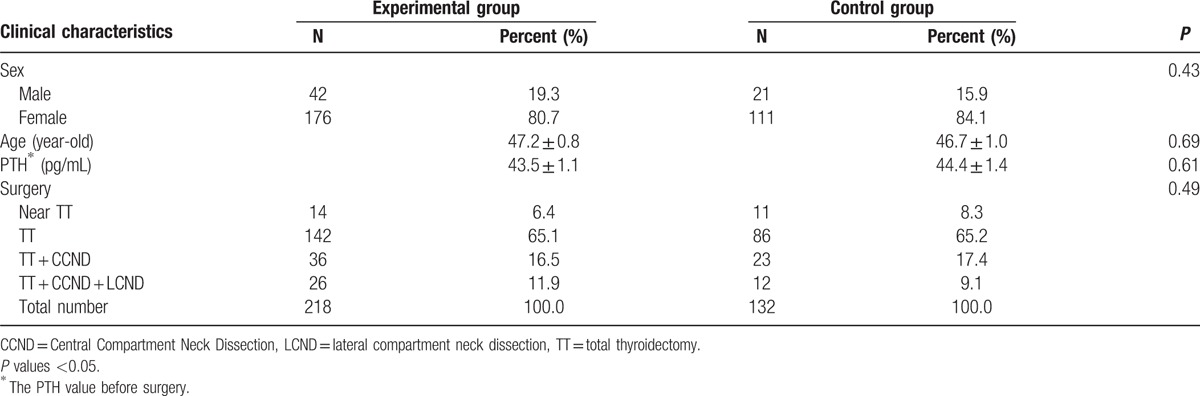
The clinical characteristics of patients, including their sex, age, and the baseline parathyroid hormone (PTH) value before surgery.

The incidence of thyroid diseases is higher among women; hence, our study sample consisted of 176 (80.7%) women and 42 (19.3%) men in the experimental group, and 21(15.9%) women and 111 (84.1%) men in the control group. In the experimental group, the median age was 47.2 ± .8 years, ranging from 14 to 72 years, and the mean value of the parathyroid hormone levels before surgery was 43.5 ± 1.1 pg/mL. In the control group, the median age was 46.7 ± 1.0 years, ranging from 14 to 73 years, and the mean value of the parathyroid hormone levels before operation was 44.4 ± 1.4 pg/mL. Total and near-total thyroidectomies (TT) constituted 71.5% (73.5%) of the cases, TT + CLND 16.5% (17.4%) of the cases, and TT + CLND + LCND 11.9% (9.1%) of the cases in the experimental (control) group.

During the surgery, we classified the types of parathyroid glands in the patients according to our new system (Table 2). The most common type was type B1, constituting 53.8% of all the parathyroid glands in our survey. Type A, often deemed as the safest type of parathyroid, was the second most common type (20.9%). There were occasional cases where no parathyroid glands were found in a patient; this phenomenon does not mean that the patient has no parathyroid gland at all, but that the patient might have ectopic parathyroid glands or type A, in which the parathyroid is not attached to the thyroid gland. The rarest type was type B3, which has the potential to become either type B2 or C; this depends on the surgeon, who decides whether total auto-transplantation is needed or not.

**Table 2:**
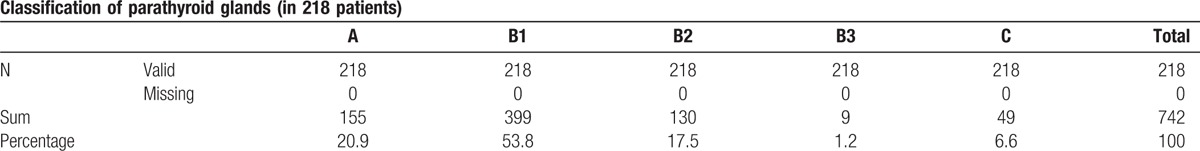
The statistics of parathyroid classification in 218 patients using the new nomenclature, expressed as number (percent). The most common type was type B1 (53.77%); the second most common type was Type A (20.89%). The rarest type is Type B3.

In the old classification system (Table 3), the upper parathyroid glands in the cricothyroid joint region and on the back of the thyroid envelope, constituted 25.8% and 62.9%, respectively, of those found on the right side of the neck. The lower parathyroid glands, most often seen on the back of the thyroid envelope (about the middle-lower 1/3 intersection point) and the ectopic type hidden in the central compartment of lymph nodes, constituted 60.6% and 6.1%, respectively. Similarly, the parathyroid glands on the left side comprised 33.3% and 55.3% of the upper parathyroid glands, and 58.3% and 13.6% of the lower parathyroid glands. The ectopic type was significantly more common on the left side than the right (*P* = 0.04), which indicates that the lower parathyroid gland on the left side of the neck is more likely to be located in the central compartment than that on the right side.

**Table 3:**
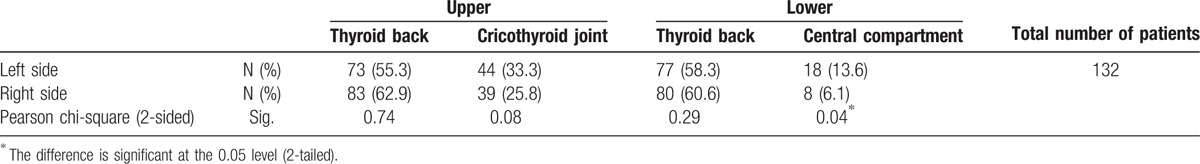
Comparison of parathyroid gland frequency with Pearson's chi-square test between left and right side.

Based on this novel classification, we decided to preserve parathyroid glands of types A and B1 in situ, while performing total auto-transplantation for type C and half autograft for type B2. As for type B3 parathyroid glands, they were either treated as type C or B2 depending on whether they could be kept in situ or not. Among our patients, 41.1% were found to have 3 parathyroid glands, and 44.3% were found to have 4 (Table 4). In the applied group, the mean number of parathyroid glands per patient was 3.4 ± 0.05, but the mean in the nonapplied group was 3.2 ± .07, which was a significant difference (*P* = 0.01) (Table 5). The total and average follow-up time was 2510 months and 7.2 months, respectively. Upon follow-up of the patients in the experimental group, 43.6% of the patients presented with transient hypoparathyroidism, whereas none presented with permanent hypoparathyroidism. In the control group, the incidence of transient and permanent hypoparathyroidism was 42.4% (*P* = 0.83) and 3.0% (*P* = 0.01), respectively (Table 6). This demonstrates unequivocally that our classification system helped reduce the rate of permanent hypoparathyroidism significantly, but there was no obvious influence on transient hypoparathyroidism. The duration of hypoparathyroidism for more than 6 months was defined as permanent hypoparathyroidism in this study. According to the correlation test among every type of parathyroid glands in our nomenclature system, types A, B1, B2, C, the central compartment type in the lower left side (as defined in the old system), and the number of parathyroid glands were correlated to the incidence of hypoparathyroidism (Table 7).

**Table 4:**
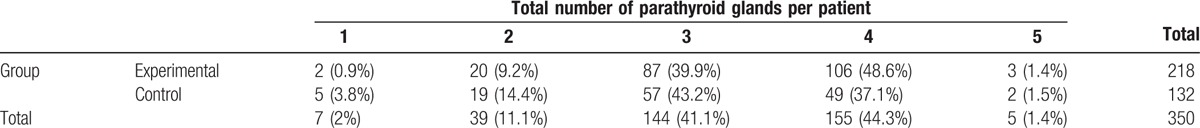
The total number of parathyroid glands per patient found with the new classification in surgery. The most common numbers of parathyroid glands found in both groups were 4 and 3.

**Table 5:**
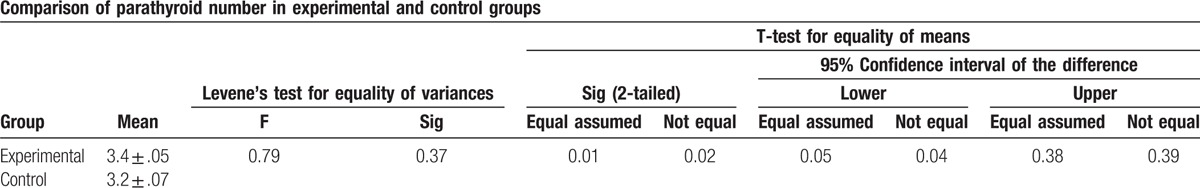
T-test for the number of parathyroid glands per patient in experimental and control groups. The related Sig (2-tailed) *P* = 0.01. A significant difference appeared in the numbers of parathyroid glands found in surgery between the 2 groups.

**Table 6:**
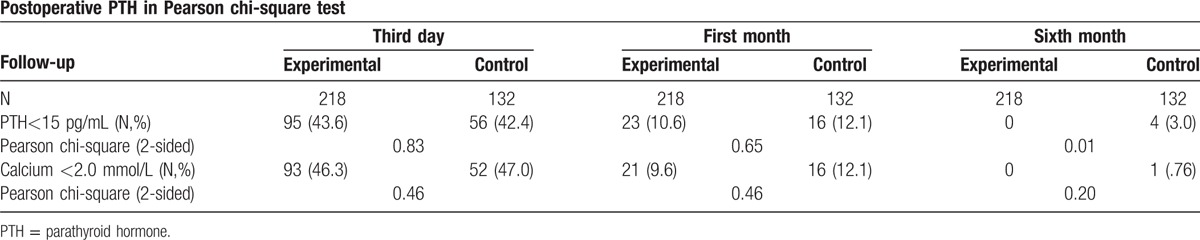
Postoperative parathyroid hormone (PTH) values, compared using the Pearson chi-square test. The PTH value was divided into 2 level: <15 pg/mL (hypoparathyroidism) and ≥15 pg/mL (normal). The calcium value was divided into 2 level: <2.0 mmol/L (hypocalcemia) and ≥2.0 mmol/L. A duration of 6 months was set as the cut-off to differentiate transient and permanent hypoparathyroidism. *P*-values < 0.05 indicate a significant difference.

**Table 7:**
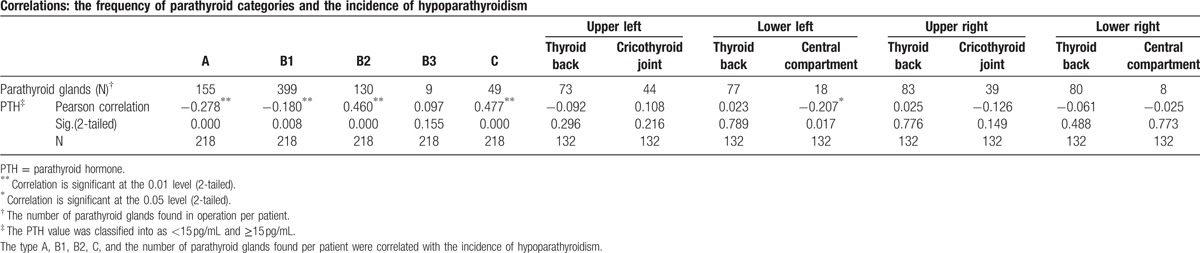
Correlations between each type of parathyroid gland and the incidence of hypoparathyroidism in the applied group.

## 4 Discussion and conclusions

### 4.1 Frequency of each type of parathyroid gland

We created a nomenclature system to define the relationship between parathyroid and thyroid glands, aiming to indicate the signs for auto-transplantation in the procedure of preserving the parathyroid in thyroidectomy. After modification and refinement, we applied it to all the patients undergoing thyroidectomy and other neck surgeries related to the parathyroid glands and it was found to be suitable for both sides of the neck. Among the 218 patients who underwent total or near-total thyroidectomy with or without central neck lymph node dissection under this novel system, the most common type of parathyroid gland was type B1 (53.8%), which retains adequate blood supply after separation from the thyroid, resulting in the normal parathyroid function and normal calcium levels. It is usually easily located, near the recurrent laryngeal nerve and Zuckerkandl Tubercle (ZT), and blood supply is visible. In our experience, it is simple to preserve this type of parathyroid gland in situ.

Another commonly found type is type A, which is not attached to the thyroid tissue; there is no need to operate on it except in the case of thyroidectomy with central lymph nodes. When there is a surgery with central lymph node dissection, the surgeon should be careful in dissecting type A parathyroid glands, and should check the operative specimen to make sure no parathyroid gland is present there, to ensure the parathyroid was not removed accidentally. As the anatomic location of the parathyroid glands (particularly the inferior parathyroid gland) varies greatly, these glands can be mistakenly identified as metastatic lymph nodes within the central neck. The rate of permanent hypoparathyroidism after total thyroidectomy and central lymph node dissection ranges from 1.4% to 14.3%.^[8]^ The accidental removal of these glands can cause transient or permanent hypoparathyroidism and hypocalcemia, which can directly affect patients’ quality of life. Therefore, effective methods to visualize additional lymph nodes while preserving the parathyroid glands are needed.

When a seemingly type A gland undergoes a color change at the end of the operation, it should be re-classified as type B2. The reasonable explanation is that there is a blood vessel entering the thyroid and coming out to supply the parathyroid gland, as is mentioned in Halsted's classification.^[9]^ Much less common in our series was the type C gland, which accounted for 6.6% of the cases we observed. Parathyroid glands of this type should be of certain concern because they often hide most of their form within the thyroid gland. Preservation of type B3 parathyroid glands in situ is more technically difficult than for other types, owing to its indistinct blood supply and general distance from the central line. There are 2 outcomes: it can be treated as type B2, to be cut in half; or as type C, to be removed from the thyroid for auto-transplantation.

### 4.2 Difference between old and new systems

Types B2, B3, and C should be auto-transplanted partially or totally. Auto-transplantation is adopted worldwide as a remedial measure to rescue the advertently dissected parathyroid. However, surgeons regularly encounter cases where permanent hypoparathyroidism occurs in spite of preservation of all the parathyroid glands in situ. A plausible explanation is that in adhering to the rule of keeping all parathyroid glands “in-situ,” even when a parathyroid gland is found to change color—a sign of devascularization—the surgeon will not choose to perform an autograft in advance, which may result in parathyroid function failure. This practice may also explain the high incidence of hypoparathyroidism with the in situ preservation of thyroid glands. If we pre-emptively auto-implant the devascularized parathyroid gland, the burden on the remaining parathyroid tissue will be decreased, and the parathyroid function is more likely to be preserved.

In our new system, the main difference is that the distal half of 1 parathyroid gland is cut into pieces to initiate auto-transplantation. Identifying the type of parathyroid gland and the appropriate operation will help predict the incidence of postoperative hypoparathyroidism and reduce the duration of medication. These parameters are mainly affected by the type of parathyroid gland, especially types B2 and B3, which need more patience during the procedure and a longer time for medication. Patients with types A and B1 tend to recover faster during the immediate postoperative period.

Furthermore, ectopic parathyroid glands in the central compartment were significantly more common on the left side of the neck than the right side under the old system, which correlated with the incidence of hypoparathyroidism. We therefore considered that CCND might elevate the chance of parathyroid damage through harming the vasculature or inadvertent excision, especially on the left side. More attention should be paid when dealing with central compartment lymph nodes, as was stressed by the conclusion of the novel system above.

### 4.3 Identification of parathyroid glands

In the initial design of this system, parathyroid glands of type B2, B3, or C would be identified via frozen section, which resulted in a positive identification rate of 99%. In clinical practice, however, this process was time- and tissue-consuming. This is especially relevant in auto-transplantation, where a shorter interval between separation from the parathyroid glands and auto-transplantation could lessen warm ischemia and raise the success rate. Next, we distinguished parathyroid glands by their oblate ellipsoid figure, clay-bank color, and the float test in water. Recently, the technology of carbon nanoparticles (CNs) has been applied to distinguish developing lymph nodes from nondeveloping parathyroid glands, which can facilitate preservation of the normal anatomic structure and physiologic function of parathyroid glands during thyroidectomy and central neck dissections.^[10]^ However, frozen sections are still a necessity when encountering an atypical parathyroid. In this study, we excluded patients in which even a frozen section could not positively identify the gland.

The inability to identify a parathyroid gland in the expected location during an operation invariably causes anxiety in surgeons. In such a case, the removed thyroid should be checked carefully to find a possible hidden parathyroid gland. After the thyroid gland is removed, the neck should be examined as well. If nothing resembling a parathyroid gland is found, there might be a type A or an ectopic parathyroid elsewhere in the body. The study demonstrated that the number of parathyroid glands found in thyroidectomy was correlated with the rate of hypoparathyroidism in both the experimental and control group. Besides, application of the novel nomenclature seemed to expose more parathyroid glands than in the control group (*P* = 0.01) according to this research. More evidence will be needed in a further study.

### 4.4 Other complications of thyroidectomies under this new system

The nomenclature system provides a guide for preserving the parathyroid glands in total or near-total thyroidectomy with or without central lymph node dissections. Keeping the parathyroid types from A through C in mind will help surgeons decide between in situ preservation and auto-transplantation. Parathyroid types B1 and B2 are typically in a close relationship with the recurrent laryngeal nerve and occasionally cross with several small vessels from the inferior thyroid arterial. Such types of glands often appear on the mid-posterior surface of the thyroid lobe. The surgeon must be aware that the presence of a parathyroid gland in this location, particularly if the small vessel is bleeding, may cover the regional anatomy, rendering the recognition of normal structures more difficult and putting the recurrent laryngeal nerve at risk. Hence, apart from providing a guideline for auto-transplantation and reducing the incidence of permanent hypoparathyroidism, the application of our classification system might reduce the incidence of other complications in thyroidectomy, such as damage to the recurrent laryngeal nerve. More observation is needed to prove these claims and to describe the role of our system in parathyroid auto-transplantation.

### 4.5 Efficacy of new system in preventing permanent hypoparathyroidism

The incidence of permanent postoperative hypoparathyroidism, defined by a hypocalcemic status and hypoparathyroid hormone level lasting more than 6 months after thyroidectomy, was 0% in the applied group, which was significantly lower than the 3.0% incidence in the nonapplied group (*P* = 0.01). This value is also lower than that reported in the medical literature; 4.4% of patients in an unselected study population developed hypoparathyroidism after bilateral operations.^[11]^ Even higher rates have been reported, especially after extensive resections. Besides, it is easier to find more parathyroid glands under the new system according to our study, which also aids in their preservation. The applausible explanation might be that our new system could remind the surgeon to be careful when dealing with all types of parathyroid glands and could find accidentally removed glands.

The results present in this study were obtained in a single center. To fully validate our new system, further studies with a larger cohort and rigorous design are warranted. Enlarged comparative studies with patients who underwent thyroidectomy before the implementation of this classification system should be performed and a longer follow-up is badly required. Some centers use 1 year as the definition of permanent hypoparathyroidism, considering that some parathyroid glands can recover within a longer time. Though we attempted to address any potential bias by screening the patients thoroughly and ensuring strict follow-ups, a more precise and accurate study might yield more credible conclusions and contribute to creating the perfect method for avoiding hypoparathyroidism after thyroidectomy. Besides, in the evaluation of parathyroid glands vitality, if the devascularization is caused by the interruption of the arterial pedicle, the gland does not become discolored, but its vitality is likewise compromised. It is not easy for surgeons to recognize the artery supply of parathyroid gland. According to our study, we suggest operating from the lateral to central side on the back of thyroid carefully to reduce the arterial damage of parathyroid glands. A further study on the arterial affection at surgery with a special technique remains to be conducted.

This novel system of classifying parathyroid glands, based on observations of their function after the thyroid is cut, was created to provide a guide for determining the correct time when a parathyroid auto-transplantation is needed in thyroidectomy (while in situ). Furthermore, the system can supplement the traditional preservation techniques. Mastering the classification can help the surgeon be more careful during operation and reach appropriate decisions between keeping the gland in situ or opting for auto-transplantation. Ultimately, the goal is to reduce the incidence of permanent hypoparathyroidism. Our new classification system of parathyroid glands, which considers the relationship between the thyroid and parathyroid glands as well as the color change after separation, has been shown to decrease the incidence of permanent hypoparathyroidism. Although we have not achieved total prevention, the novel nomenclature system gives us hope that in the future, this complication can be fully controlled.
